# Response diversity is a major driver of temporal stability in complex food webs

**DOI:** 10.1098/rspb.2025.0730

**Published:** 2025-11-05

**Authors:** Alain Danet, Sonia Kéfi, Thomas F. Johnson, Andrew P. Beckerman

**Affiliations:** ^1^ School of Biosciences, Ecology and Evolutionary Biology, University of Sheffield, Sheffield S10 2TN, UK; ^2^ ISEM, Univ Montpellier, CNRS, IRD, Montpellier 34070, France; ^3^ Santa Fe Institute, Santa Fe, NM USA

**Keywords:** food web, temporal stability, response diversity, diversity-stability, environmental stochasticity, theoretical modelling

## Abstract

Global change constitutes a major threat to biodiversity and ecosystem functions and places the temporal stability of ecological communities at risk. Classic theory identifies species richness and food web structure as key drivers of temporal stability, while recent work highlights response diversity—variation in species’ responses to environmental perturbations—as a critical stabilizer via asynchrony in population fluctuations. However, how these mechanisms interact in complex, multi-trophic communities remain unresolved. Using a stochastic, bioenergetic food web model, we integrate these multiple bodies of theory to reveal that response diversity is a major driver of community stability. Moreover, our integrated theory reveals that positive stability-richness relationships emerge only in the presence of response diversity. In contrast to previous work, we also find that food web structure is only a secondary driver of community stability but interacts with response diversity to determine the sign of the stability-richness relationship. Our study reveals identifiable pathways by which food web structure and response diversity drive community stability and raises concerns about how the loss of response diversity may lead to a breakdown of community stability.

## 1. Introduction

Understanding how ecological communities withstand environmental perturbations and thus remain stable is a central goal in ecology [1,2]. Theory and experiments show that species diversity enhances temporal stability by buffering perturbations [3–11]. This stability arises from two components: (i) population-level stability, which measures the temporal variability in biomass averaged over all species in the community; and (ii) asynchrony, which captures the degree to which species’ fluctuations offset one another [8,12]. Asynchrony underpins diversity’s stabilizing effects [3,6–8,12,13] and is hypothesized to be mainly driven by response diversity, the diversity of species’ responses to environmental fluctuations [14–17].

Our understanding of the mechanisms driving temporal stability and stability-richness relationships remains largely restricted to simplified assemblages such as competitive [3–8] and plant–herbivore systems [9–11]. This is problematic as complex assemblages like food webs may behave differently as trophic interactions create strong interdependence in species’ dynamics [18–22]. Multiple lines of evidence support that trophic interactions can alter community stability [23–26] through population stability [2,20,27–29] and asynchrony, and that food web structure can interact with response diversity and species richness, the main drivers of temporal stability [20–22]. In the following paragraphs, we review the evidence around this array of theory and then present our *in silico* experiment aiming to understand how these mechanisms combine to simultaneously affect temporal stability of complex, multitrophic, ecological communities.

Stability at the population level is a key component of community stability. Previous theoretical and empirical studies highlighted that population stability decreases strongly with environmental stochasticity, while species richness can have mixed effects, where a rise in richness has been observed to both decrease and increase population stability [30–32]. Food web structure can also mediate these effects as higher trophic levels may stabilize populations via lower mortality [23,33] or top-down control [24], while strong interactions and high connectance often decrease population stability [2,20,27,29]. Crucially, these population responses to community structure are further mediated by asynchrony to shape community stability [8,12].

This asynchrony emerges from two pathways in the absence of strong demographic stochasticity [31,34]: (i) response diversity to environmental changes and (ii) species interactions. Response diversity, arising from differences in species’ niches and environmental preferences, leads to varied reactions to the same environmental perturbations [14,16]. For example, within a community, some species may increase in abundance under warmer conditions while others decline, depending on their thermal tolerances [35]. Using a trait-based or non-trait-based approach, previous studies showed that response diversity drives asynchrony [7,12,15], and thereby stability [3,7,12].

Response diversity modulates asynchrony through two distinct mechanisms: portfolio effects and compensatory dynamics. Portfolio effects occur when species exhibit independent responses to environmental fluctuations, leading to statistical averaging that stabilizes aggregate community biomass, thereby increasing community stability [4]. This effect represents statistical averaging weighted by response diversity and is strongest when species’ environmental responses are uncorrelated and weakest when response diversity is low (i.e. when species’ responses are positively correlated). Compensatory dynamics, by contrast, arise when species show negatively correlated responses, providing additional stabilization [8]. Empirical work in grasslands has demonstrated that portfolio effects—driven primarily by response diversity—constitute the dominant mechanism behind diversity’s stabilizing effects [4,8].

Originally defined as the diversity of species responses within functional groups [14], the role of response diversity on asynchrony via portfolio effects and compensatory dynamics in multi-trophic systems remains poorly understood. Theory suggests that trophic interactions might alter the effects of response diversity on asynchrony, either synchronizing prey dynamics through top-down control [18,19] or enhancing compensatory dynamics via prey switching and trophic cascades [20–22]. These food web structures might influence stability-richness relationships, where for example, species-rich communities often contain more trophic levels. This makes it challenging to disentangle the effects of richness from those of food web structure [25,26,29] *per se* [36,37].

To reconcile this wide range of theory defined across multiple ecological scales and decompose the relative contribution of each process to stability and stability-richness relationships, we here integrate these sets of processes into an extended bioenergetic food web model [23,38] that incorporates environmental stochasticity and response diversity [39–41]. We investigate relationships among multiple processes across 45 000 *in silico* communities with varying levels of species richness, trophic complexity, connectance, and where we experimentally manipulate interaction strengths, response diversity and environmental stochasticity. This *in silico* experiment allowed us to specifically evaluate whether response diversity, species richness and food web structure act additively or interactively to drive community stability, and under what conditions these factors lead to positive or negative stability-richness relationships. Finally, using a structural equation model and linear models, we test how the factors linked to environmental fluctuations and their buffering (environmental stochasticity and response diversity) and the ones mediating them (species richness and food web structure) affect the components of community stability (population stability and asynchrony).

## 2. Methods

### (a) Bioenergetic model

We simulated the dynamics of complex food webs using the allometric, bioenergetic model [23,38,42,43]. This model is widely used to simulate the dynamics of complex ecological communities because of the simplicity of its parametrization, where metabolism, growth and foraging rates are based on species body mass and metabolic types using the metabolic theory of ecology [44]. The model describes species biomass ($B_{i}$) dynamics over time of the primary producers ($B_{i\in \{prod.\}}$, equation (2.2)) and the consumers ($B_{i\in \{cons.\}}$, equation (2.1)):


(2.1) $$\frac{dB_{i\in \{cons.\}}}{dt}=\sum_{j\in \{res.{\}}_{i}}x_{i}y_{i}B_{i}F_{ij}-\sum_{j\in \{cons.{\}}_{i}}\frac{x_{j}y_{j}B_{j}F_{ji}}{e_{ij}}-x_{i}B_{i}-d_{i}B_{i},$$

(2.2) $$\frac{dB_{i\in \{prod.\}}}{dt}=r_{i}B_{i}G_{i}-\sum_{j\in \{cons.{\}}_{i}}\frac{x_{j}y_{j}B_{j}F_{ji}}{e_{ij}}-d_{i}B_{i}.$$

The consumers gain biomass by consuming their resources, where $j\in \{res.{\}}_{i}$ are the resources of the consumer i, at a rate that depends on their metabolic rate ($x_{i}$), maximum consumption rate ($y_{i}$) and on the functional response ($F_{ij}$) describing how the rate of consumption of a consumer i on one of its resources j vary with the biomass of this resource. The consumer metabolic rate was derived from ectotherm mass-specific metabolic rate ($a_{x}=0.88$; electronic supplementary material, table S1) and species body masses ($M_{i}$): $x_{i}=a_{x}M_{i}^{b}$, with b=−1/4 as defined in the allometric theory of metabolism [23,44].

Species then lose biomass by being consumed, where $j\in \{cons.{\}}_{i}$ are the consumers of the species i, $e_{ij}$ is the assimilation efficiency of the consumer j on the resource i [23,42,43]. Consumers also lose biomass over time through metabolic losses ($-x_{i}B_{i}$) and natural mortality ($-d_{i}B_{i}$). The primary producers gain biomass over time with a growth rate ($r_{i}$, equation (2.2)) and a functional response ($G_{i}$) describing how the growth of the producers varies with their biomass. They lose biomass by being consumed by consumers and by natural mortality.

The growth of the primary producers (equation (2.3)) is logistic:


(2.3) $$G_{i}=(1-\frac{\sum_{j\in \{prod.\}}\alpha_{ij}B_{j}}{K_{i}}),$$

where the growth rate is maximal when $\sum_{j\in \{prod.\}}\alpha_{ij}B_{j}$ approaches 0 and null when it approaches $K_{i}$. $\alpha_{ij}$ is the per capita effect of the producer j on the producer i, $K_{i}$ is the carrying capacity for the producer j. The feeding rate of a consumer feeding on a resource (equation (2.4)) is described by a functional response:


(2.4) $$F_{ij}=\frac{\omega_{ij}B_{j}^{h}}{B_{0}^{h}+c_{i}B_{i}+\sum_{k\in \{res.{\}}_{i}}\omega_{ik}B_{k}^{h}},$$

which depends on the relative preference of the consumer on the resource ($\omega_{ij}$) is limited by a half-saturation rate ($B_{0}$), intraspecific interference coefficient ($c_{i}$) and on the availability of its other resources ($\sum_{k\in \{res.{\}}_{i}}\omega_{ik}B_{k}^{h}$). Finally, the h exponent controls the shape of the functional response from a saturating function (h=1, Holling type II) to a sigmoid (h=2, Holling type III).

### (b) Environmental stochasticity and response diversity

We added a stochastic natural mortality rate to the species dynamics, representing a fluctuating environment. Based on the literature and previous models [33,39], we assumed that natural mortality rates scales inversely with the species body mass ($d_{i}=d_{0}M_{i}^{-1/4}$), as all other physiological parameters. Following previous work, we used a basal natural mortality rate of $d_{0}=0.4$ [39].

Introducing environmental stochasticity via mortality rates allows to consider stochasticity without modifying species interactions themselves [39]. The stochastic part of the mortality rates ($\epsilon_{i,t}$) follows a normal distribution 0 centred ($\epsilon_{i,t}∼N(0,\sigma_{e}^{2})$) with a variance ($\sigma_{e}^{2}$). The mortality rates of each species at the time t equals to $d_{i,t}=d_{i}e^{\epsilon_{i,t}}$, which ensured that the mortality rates never became negative [39]. We simulated ϵ in two steps: we first simulated a temporally correlated Ornstein–Uhlenbeck process to generate the stochastic part of species mortality rates ($a_{i,t}$) and then we multiplied those values by a correlation matrix to control the level of response diversity. The Ornstein–Uhlenbeck process is a modified Brownian motion where the stochastic values tend to come back to the central value (i.e. $a_{i}=0$) such as $a_{i,t}$ varies according to this stochastic differential equation: $da_{i}=(0-a_{i})d_{t}+\sigma_{e}dW_{i}$, where $dW_{i}$ is a Brownian motion. The strength of environmental stochasticity was then controlled by $\sigma_{e}$.

Response diversity was controlled by the correlation among species stochastic mortality rates ($\rho_{ij,i\neq j}$) such that response diversity is maximal when the stochastic component of species mortality rates ($\epsilon_{i,t}$) is uncorrelated (ρ=0), and response diversity is null when the stochastic component of species mortality rates is perfectly correlated (ρ=1) [39,41,45]. Formally, the species mortality rates were a product of the stochastic mortality rates generated by the Ornstein–Uhlenbeck process and a variance-covariance matrix such as:


(2.5) $$\begin{array}{r} \epsilon_{i,t}=\left[\begin{array}{cccc} \rho_{11}\sigma_{e}^{2} & \rho_{12}\sigma_{e}^{2} & \cdots & \rho_{1n}\sigma_{e}^{2}\\ \rho_{21}\sigma_{e}^{2} & \rho_{22}\sigma_{e}^{2} & \cdots & \rho_{2n}\sigma_{e}^{2}\\ \vdots & \vdots & \ddots & \vdots \\ \rho_{n1}\sigma_{e}^{2} & \rho_{n2}\sigma_{e}^{2} & \cdots & \rho_{nn}\sigma_{e}^{2} \end{array}\right]\left[\begin{array}{c} a_{1,t}\\ a_{2,t}\\ \vdots \\ a_{n,t} \end{array}\right], \end{array}$$

where $a_{i,t}$ is the stochastic value generated by the Ornstein–Uhlenbeck process at time t, $\rho_{ii}=1.0$ while all $\rho_{ij,i\neq j}$ were equal and control the level of response diversity. All interspecific correlations being equal, we assume no structure in response diversity across the trophic levels, which can contrast with the original definition that considered response diversity as the diversity of species responses within functional groups [14].

### (c) Simulation design

We generated food web structure using the niche model [46] with an initial species richness from 10 to 60, and connectance from 0.02 to 0.38 (electronic supplementary material, table S1). We required food webs without cannibalistic links and fully connected, i.e. we discarded food webs that contained disconnected species.

We varied the strength of environmental stochasticity by varying $\sigma_{e}$ from 0.1 to 0.6, the range of mortality rates observed in protists [39]. We decreased response diversity by increasing ρ from 0 to 1 (i.e. response diversity is 1−ρ; electronic supplementary material, figure S1).

We varied the body mass structure of the food web to drive variation in trophic interaction strength and mortality rates, because metabolic and mortality rates ($x_{i}$ & $d_{i}$) scale with species body mass. To do so, we varied the predator–prey mass ratio (Z) from 1 to 100 (electronic supplementary material, figure S1b). Species body masses ($M_{i}$) were computed as: $M_{i}=Z^{TL_{i}-1}$, where ${TL}_{i}$ is the trophic level of species i. We set h=2, i.e. a functional response of type III (equation (2.4)), and varied predator interference (i.e. from c=0 to c=1, equation (2.4)), as predator interference has been shown to have a tremendous role on population stability [23].

In the absence of environmental stochasticity and with a type III functional response, species dynamics reach a steady state without visible oscillations. We set uniform consumer preferences for their n resources such as $\omega_{ij}=1/n$, half-saturation rate to $B_{0}=0.5$, the maximum consumption rate to $y_{i}=4$, $r_{i}=1.0$ based on previous studies [23]. Intraspecific competition was set to unity ($\alpha_{ii}=1.0$) and interspecific competition ($\alpha_{ij,i\neq j}$) to 0.5. We set a global carrying capacity of $K^{\prime }=10$ to ensure that consumers were not limited by the biomass input from primary producers. We standardized the carrying capacity by the number of primary producers and by the interspecific competition among producers to ensure that the effects of species richness on temporal stability are not driven by the increase in the number of primary producers, thereby trivially increasing biomass input for consumers. We then standardized $K^{\prime }$ such as $K_{i}=\frac{1+\alpha_{ij}(S_{prod.}-1)}{S_{prod.}}K^{\prime }$ [28], where $S_{prod.}$ is the number of primary producer species. However, we found that our results were not affected when removing the standardization of carrying capacity (electronic supplementary material, figure S2).

We numerically solved the stochastic differential equation system during at least 2000 timesteps and collected the last 500 timesteps, similarly to previous studies [23]. We numerically solve the system with $\Delta_{t}=0.1$ or $\Delta_{t}=0.05$ when instability was detected during the simulations because of stiff changes in biomass. We set extinction threshold to $10^{-6}$ and if an extinction happened in the last 500 timesteps, we continued to run the simulation for another 1000 timesteps until there was no extinction events during the last 500 timesteps. When we found disconnected species at the end of the simulation (i.e. a primary producer without consumer or a consumer without prey), we set their biomass to 0 and re-ran the simulation for another 1000 timesteps. Except in the case of disconnected species, we re-ran simulations with species starting biomass equal to their biomass at the last timestep of the previous simulation. Supplementary analysis showed that alternative choices of simulation processes, such as longer simulations, rebuilding food webs (i.e. resetting consumer preferences, recomputing species body mass according to the new food web) after removing disconnected species did not affect our results (electronic supplementary material, figure S2). The food web generation and simulations were done in Julia by developing an extension of the package EcologicalNetworksDynamics.jl [43].

In summary, we generated 46 880 simulations over a wide range of food web structure (median (5%, 95%), species richness: 13.0 (5.0,26.0), connectance: 0.12 (0.058,0.3), average interaction strength: 0.02 (0.0031,0.071), maximum trophic level: 2.5 (2.0,3.5)) (electronic supplementary material, table S2).

### (d) Simulation outputs

We measured food web properties that have been linked with stability in the literature. We measured total biomass, species richness, connectance, average trophic level weighted by biomass, average omnivory, and average interaction strength. Food web properties were measured at each of the 500 timesteps and then averaged, except connectance and trophic level that were static. Connectance was computed as C=L/S(S−1), where L is the number of trophic links and S is the number of species in the community. Species trophic level was computed recursively from the bottom of the food web, such as the trophic level of a consumer was equal to the average trophic level of its resources added to 1, the trophic level of primary producers was set to 1 [47]. Average trophic level ($\hat{wTL}$) was then equal to: $\hat{wTL}=\sum_{i}1/\hat{B_{i}}\times {TL}_{i}$, where ${\mathrm{TL}}_{i}$ is the trophic level and $\hat{B_{i}}$ the average biomass of the species i. The degree of omnivory of a consumer was computed such as the sum of squares of its resource trophic levels weighted by the relative preference of the consumer for each resource: $\sum_{i}({\mathrm{TL}}_{i}-\hat{\mathrm{TL}}{)}^{2}\omega_{i}/\sum \omega_{i}$ [47]. Interaction strength was quantified as the biomass fluxes going from every consumer/resource pair, such as: $I_{ij}=x_{i}y_{i}B_{i}F_{ij}$ (equation (2.1)), and averaged over time. Our way of measuring interaction strength is very similar to previous metrics used in previous studies with the difference here that we average fluxes over time and not the maximal interaction strength [20]. Finally, the average interaction strength of a community was computed as the average of the S×S interaction strength matrix at the exclusion of null interaction strengths (i.e. excluding absence of trophic interactions).

We assessed the effects of food web structure, environmental stochasticity and response diversity on the temporal stability of community biomass ($S_{com}$). We measured temporal stability of biomass as the inverse of the coefficient of variation. We further partitioned stability into population stability ($S_{pop}$) and asynchrony (ϕ) as:


(2.6) $$\begin{array}{r} S_{com}=S_{pop}\times \phi , \end{array}$$

with $S_{com}=\mu_{tot}/\sigma_{tot}$, $S_{pop}=\mu_{tot}/\sum_{i}\sigma_{i}$, $\phi =\sum_{i}\sigma_{i}/\sigma_{tot}$; where $\mu_{tot}$ and $\sigma_{tot}$ are, respectively, average total biomass and standard deviation of total biomass [8,12].

To obtain further mechanistic insight about the effects of environmental stochasticity, response diversity and food web structure on stability, we partitioned asynchrony into portfolio effects (PFE) and compensatory dynamics stemming from species interactions [8] ($CPE_{int}$). The measurement of portfolio effects has been a topic of debate [8,48]. Doak’s definition [4] defines portfolio effects as the product of statistical averaging effects (SAE) and compensatory effects arising from response diversity [4,8] ($CPE_{env}$). It then quantifies the portfolio effect ($PFE=SAE\times CPE_{env}$) emerging from independent species fluctuations, i.e. the statistical averaging effect (SAE), weighted by the effects of response diversity of species to environmental fluctuations, such as portfolio effects are dampened if species have correlated responses to environmental fluctuations. To compute portfolio effects and compensatory dynamics stemming from species interactions, we (i) partition asynchrony into statistical averaging effect and compensatory dynamics (i.e. ϕ=SAE×CPE, equation (2.7)); and (ii) partition compensatory dynamics originating from species interactions ($CPE_{int}$) and from response diversity ($CPE_{env}$).


(2.7) $$\begin{array}{rl} S_{com} & =S_{pop}\times SAE\times CPE\\ S_{com} & =S_{pop}\times SAE\times CPE_{int}\times CPE_{env} \end{array}.$$

SAE is the share of asynchrony assuming that species are independent, meaning that the total variance of the community is equal to the sum of species variances only (i.e. species covariances are null). We can define community stability ($S_{com,IP}$) in that scenario and then derive SAE:


(2.8) $$\begin{array}{rl} S_{com,IP} & =\frac{\mu_{tot}}{\sqrt{\sum_{i}\sigma_{i}^{2}}}=SAE\times S_{pop}\\ SAE & =\frac{S_{com,IP}}{S_{pop}}=\frac{\sum_{i}\sigma_{i}}{\sqrt{\sum_{i}\sigma_{i}^{2}}} \end{array}.$$

Compensatory dynamics in the food webs is then the remaining part of asynchrony:


(2.9) $$\begin{array}{rl} CPE & =\frac{S_{com}}{SAE\times S_{pop}}=\frac{\sqrt{\sum_{i}\sigma_{i}^{2}}}{\sigma_{tot}} \end{array}.$$

Compensatory effects can arise from predator–prey interactions ($CPE_{int}$) or from differential response of species to environmental stochasticity ($CPE_{env}$). We computed $CPE_{env}$ on the part of biomass fluctuations that are due to environmental stochasticity and response diversity ($B_{d,i}$, equation (2.10)), i.e. from the deterministic and stochastic parts of mortality rates. To do so, we used the time × species matrices of species biomass B and stochastic mortality rates E:


(2.10) $$\begin{array}{rl} B_{d,i} & =B\times diag(d_{i})\times e^{E}. \end{array}$$

We computed $CPE_{env}$ using equation (2.9) with the time x species matrix ($B_{d,i}$), so that compensatory effects owing to environmental fluctuations are mainly determined by response diversity (electronic supplementary material, figure S3). Then compensatory effects owing to species interactions were computed as: $CPE_{int}=CPE/CPE_{env}$. Finally, portfolio effects (PFE) were computed as: $PFE=SAE\times CPE_{env}$ and compensatory effects owing to species interactions as $CPE_{int}$. The final stability decomposition reads as follows:


(2.11) $$\begin{array}{r} S_{com}=S_{pop}\times PFE\times CPE_{int} \end{array}.$$

### (e) Statistical analysis

**Figure 1.**
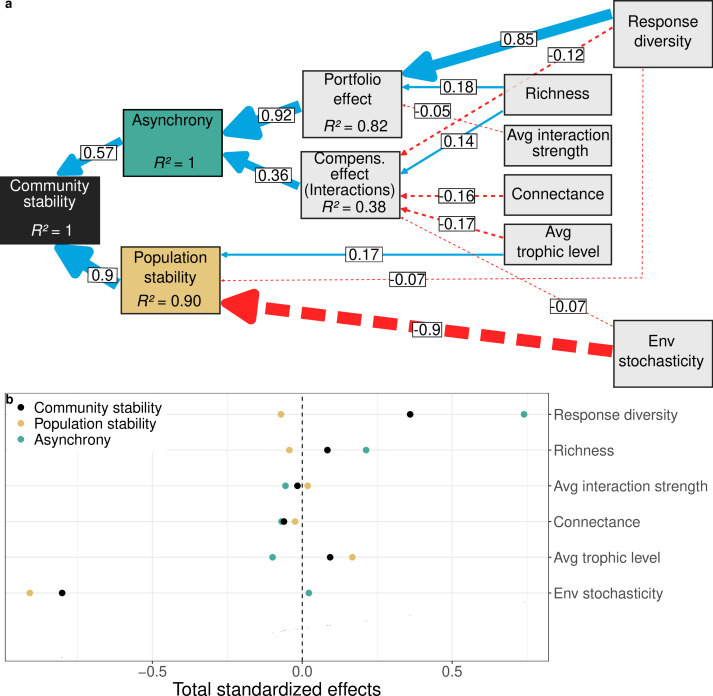
Structural equation model linking food web structure, environmental stochasticity and response diversity to the temporal stability of community biomass. (a) Continuous blue arrows display positive and dashed red arrows display negative standardized effects, the arrow widths are proportional to the absolute values of the standardized effects. Only the standardized coefficients whose absolute values are greater to 0.05 are displayed. The full table of coefficients including control variables is displayed in the electronic supplementary material, table S5. (b) Total effects of food web structure, environmental stochasticity and response diversity on community stability, asynchrony and population stability derived from the structural equation model. *n* = 46 880 simulations.

Using a structural equation model (SEM), we estimate the contribution to temporal community stability of the numerous processes reviewed in the introduction across the multiple ecological scales at which they occur (figure 1).

The arrangement of the SEM was defined *a priori* based on the logic of what ecological scales each process operates on, and on expectations from previous theoretical studies. Working from right to left in figure 1, we first specify response diversity and environmental stochasticity which operate across all species in a community. Richness, average interaction strength, connectance and average trophic level then represent structural properties of the community (food web). Moving further left, the portfolio effect and compensatory effects owing to species interactions represent aggregated species-specific contributions to processes that underpin asynchrony. Here, asynchrony is paired with population stability, also an aggregated process at the species scale.

The flow from right to left represents expectations from theory. Theory suggests a strong influence of average trophic level, average interaction strength and connectance on population stability and asynchrony [20–22,27], directly for population stability but mediated by portfolio and compensatory effects for asynchrony [8]. The SEM then reflects expected impacts of environmental stochasticity and response diversity on population stability and asynchrony partitions. Furthest left, our SEM also captures the core partition of asynchrony into compensatory effects generated by species interactions ($CPE_{int}$) and portfolio effects stemming from statistical averaging and compensatory effects owing to response diversity ($SAE\times CPE_{env}$), and from asynchrony and population stability to community stability.

Although not shown in figure 1, we included the predator–prey mass ratio and predator interference values as control variables as they have been shown to influence population stability [23]; and while average omnivory is also expected to increase population stability [27], it was not included because of strong collinearity (i.e. measured by the variance inflation factor) with average trophic level.

Prior to evaluating hypotheses in the SEM, we logged all the stability components to transform their relation from multiplicative to additive (equation (2.7)). We ensured that all the linear models composing the structural equation model presented low multicollinearity (variance inflation factor < 3; electronic supplementary material, table S3), although interaction strength and species richness were highly correlated (electronic supplementary material, figure S4). The SEM was evaluated using the R package PiecewiseSEM [49]. We computed the sum of the direct and indirect effects using the R package semEff [50]. In the main text, we reported standardized coefficients which were obtained by scaling the coefficients by the standard deviation of the response and predictor variable. Finally, we reported only the direct standardized coefficients with an absolute value equal or above 0.05, because almost all effects were statistically significant given the high numbers of simulations. These direct standardized coefficients reflect partial correlation coefficients, while total standardized coefficients reflect correlation coefficients [51].

In the second analysis, we used linear mixed-effect models to test how food web structure, response diversity and environmental stochasticity modulate stability-species richness relationships. We modelled temporal stability of community biomass according to food web structure: species richness, weighted average trophic level, connectance and average interaction strength, as well as environmental stochasticity and response diversity. We further included the two-way interactions between species richness and food web structure, between species richness and response diversity, between species richness and environmental stochasticity. Finally, we added the three-way interactions between species richness, food web structure and response diversity, between species richness, food web structure and environmental stochasticity. We included predator interference, predator–prey mass ratio as control variables and added the ID of the food web as a random intercept.

**Figure 2.**
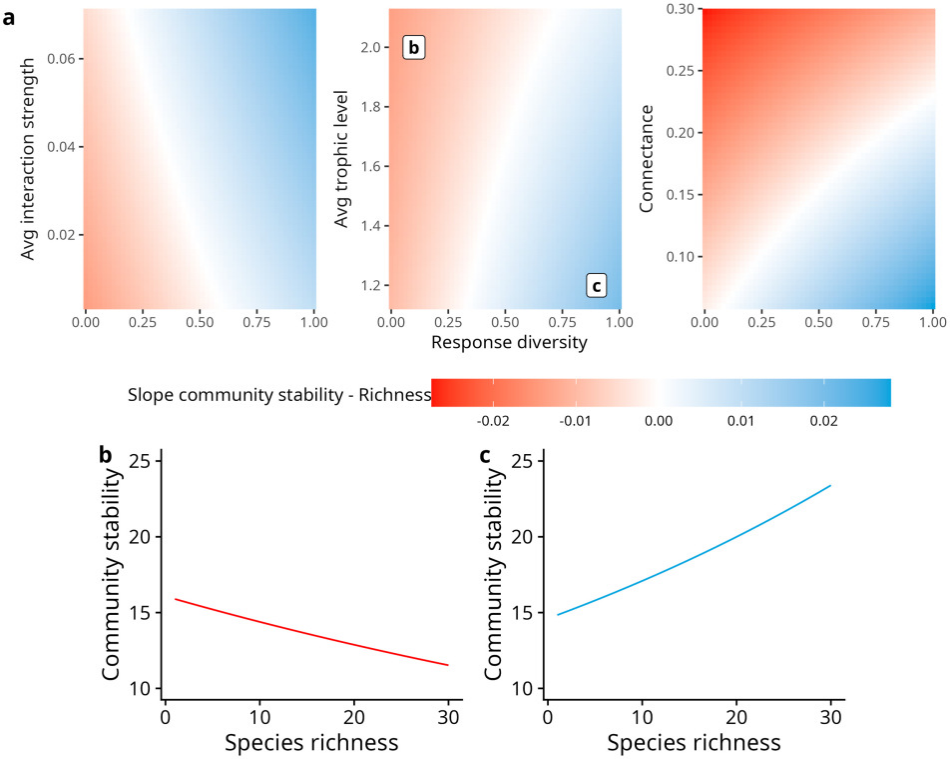
Slope of the community stability–species richness relationship based on food web structure and response diversity. (a) Diagrams displaying the slope coefficients of the effect of species richness on community stability. The average values of the control variables were used to generate the predictions: connectance = 0.14, average interaction strength = 0.025, average trophic level = 1.49, predator interference = 0.5, predator–prey mass ratio = 33, and environmental stochasticity = 0.3. (b,c) Examples of predictions from the model for two combinations of average trophic level and response diversity (values displayed in (a)). Lines display the mean predictions of the model. The coefficients of the linear model are displayed in the electronic supplementary material, figure S4. Relationships between population stability, asynchrony and species richness are displayed in the electronic supplementary material, figure S7.

We used this model to predict the shape of the relationship between community stability and species richness along a gradient of response diversity and of food web structure. To do so, we summed all the coefficients involving species richness in the model, i.e. the one-, two- and three-way terms. The values of the other variables were set to the values indicated in figure 2. The coefficients of the models are displayed in the electronic supplementary material, figure S5. The Variance Inflation Factors were lower than 3, indicating low multicollinearity (electronic supplementary material, table S4). We checked the distribution of the residuals (electronic supplementary material, figure S6) using the DHARMa R package. The model was implemented using the R package glmmTMB [52].

## 3. Results

### (a) The drivers of community stability

Applying a structural equation model on the outcomes of food web simulations revealed that the temporal stability of community biomass is positively influenced by both population stability and asynchrony, and that population stability has nearly double the contribution of asynchrony (resp. $r_{\delta }$= 0.9 and 0.57, $r_{\delta }$ is the standardized coefficient; figure 1a). This result might be driven by the relative variance of environmental stochasticity and response diversity explored in our simulations, since a higher variance in environmental stochasticity relative to response diversity would result in a higher effect size in population stability than in asynchrony because those coefficients were expressed relative to the standard deviation of the variables (i.e. standardized coefficients). However, the standard deviation of environmental stochasticity was two times lower than response diversity (resp. 0.17 versus 0.35), so that the higher importance of population stability relative to asynchrony that we report cannot be explained by the explored parameter ranges in the study. All the tested causal relationships are presented in the electronic supplementary material, table S5.

We further found that asynchrony is driven more by portfolio effects than by compensatory dynamics (resp. $r_{\delta }$= 0.92 and 0.36). In more details, response diversity has strong positive effects on asynchrony, mainly through portfolio effects ($r_{\delta }$ = 0.85), at the expense of compensatory dynamics ($r_{\delta }$ = −0.12). All other studied properties were found to have much weaker effects on asynchrony. Food web structural properties—namely average trophic level, connectance and average interaction strength—have consistent negative effects on asynchrony, through both portfolio and compensatory effects (figure 1a). Indeed, as average trophic level and connectance increase, compensatory effects are reduced (resp. $r_{\delta }$= −0.17 and −0.16; figure 1a), while average interaction strength has a small negative effect on portfolio effects ($r_{\delta }$ = −0.05). Species richness has a positive effect on both portfolio and compensatory effects (resp. $r_{\delta }$= 0.18 and 0.14). Finally, as expected by the standardization of asynchrony to total community variance (see Methods), environmental stochasticity has little effect on asynchrony.

Focusing on population stability, environmental stochasticity was found to have a strong negative effect on it ($r_{\delta }$ = −0.91). All other studied properties were found to have little effect on population stability, except for the average trophic level, which has a small positive effect ($r_{\delta }$ = 0.17; figure 1a; but see the electronic supplementary material, figure S2) and response diversity, a small negative effect ($r_{\delta }$ = −0.07).

Deriving total effects from the sum of direct and indirect effects in the structural equation model (see §2), we showed that both environmental stochasticity and species’ response diversity have by far the strongest total effects on community stability (resp. $r_{\delta }$= −0.8 and 0.36; figure 1b). Species richness and average trophic level have similar small but positive total effects on community stability ($r_{\delta }$ = 0.08), but through opposite pathways. While species richness increases community stability through a strong positive effect on asynchrony ($r_{\delta }$ = 0.21), it also weakly decreases population stability ($r_{\delta }$ = −0.04). By contrast, average trophic level increases community stability through strong positive effect on population stability ($r_{\delta }$ = 0.17) but is dampened by a negative total effect on asynchrony ($r_{\delta }$ = −0.1). Interestingly, average interaction strength also has a positive effect on population stability and a negative one on asynchrony (resp. $r_{\delta }$= 0.02 and −0.06). This results in a total negative effect on community stability ($r_{\delta }$ = −0.02). Connectance, instead, has a negative effect on community stability ($r_{\delta }$ = −0.06) through negative effects on both asynchrony and population stability (resp. $r_{\delta }$= −0.07 and −0.02).

### (b) The context dependence of stability-richness relationships

We further assessed how environmental stochasticity and response diversity interacted with food web structural properties to determine the sign of the stability-richness relationship. We found that there are strong interactions between response diversity and food web structure, which jointly determine the sign of the stability-richness relationship.

In the absence of response diversity, we found only negative stability-richness relationships, regardless of the food web structure (figure 2a). Response diversity enables the rise of positive stability-richness relationships, enhanced by average interaction strength and dampened by average trophic level and connectance. Higher interaction strength leads to positive stability-richness relationships for lower values of response diversity, and interaction strength enhances asynchrony-richness relationships (electronic supplementary material, figure S7), so that the stronger positive stability-richness relationships are found at both high interaction strength and high response diversity levels. By contrast, higher average trophic level and connectance both lead to negative stability-richness relationships, because they drive more negative population stability-richness relationships and weaker positive asynchrony-richness relationships (electronic supplementary material, figure S7).

Overall, our results highlight a contrast between the relatively small effects of food web structural properties on overall community stability (figure 1b) but their strong effects on the slope of the stability-richness relationship (figure 2a). A contrast which is consistent when considering interactive effects among food web structure, response diversity and environmental stochasticity (electronic supplementary material, figure S3).

## 4. Discussion

With projected changes in climate and land use, and associated loss of diversity, change in community structure, and potential homogenization, it is important to understand the mechanisms which allow ecological communities to remain stable despite perturbations. Recent research has focused on either the average stability of the populations composing the community or on the asynchrony of the population fluctuations [8,12]. Moreover, the majority of this work has been done in simplified assemblages, such as single-trophic-level communities. In this focused and often simplified context, community stability has been shown to be driven by asynchrony in species fluctuations, rather than by population stability [13].

However, whether this inference holds for more complex, trophically structured ecological communities such as food webs remains largely unknown. Here, we tackled this question using simulations of complex, stochastic food web dynamics via a bioenergetic model. In doing so, we reveal not only the relative contribution to temporal stability of multiple processes spanning multiple ecological scales but also predictions about the context dependency of diversity-stability relationships amidst rapid global environmental change.

Our results show that, in complex food webs, community stability is more driven by population stability than by asynchrony, in contrast with what was observed in single-trophic-level communities. As we showed above, this was not owing to the ratio of variance between environmental stochasticity and response diversity. Our study further reveals that the main drivers of community stability are environmental stochasticity and response diversity, respectively acting on population stability and asynchrony. Furthermore, despite previous work in simple communities suggesting that food web structural properties drive stability, we found that these properties have much weaker effects in more complex food webs, a result consistent across a diversity of methodological choices (electronic supplementary material, figure S2).

However, structural properties are central to our understanding of stability-richness relationships. They were found to play an important role in the sign of the stability-richness relationship, which is strongly mediated by the interaction between food web structure and response diversity. Our results highlight the dual importance of response diversity to improve our understanding of stability *per se* and of richness-stability relationships in structured ecological communities facing variable environments. In the following sections, we deliver more nuanced insights into these conclusions.

### (a) Environmental stochasticity and response diversity are the main drivers of community stability

We found environmental stochasticity and response diversity to be the main overall drivers of community stability. While environmental stochasticity decreases population stability, response diversity generates asynchrony, thereby buffering environmental stochasticity. This result is in accordance with previous theoretical and empirical findings in competitive communities [6,7,12,28,29,34]. In turn, response diversity was found to operate on asynchrony. This is in agreement with empirical findings in competitive assemblages where response diversity was quantified with trait-based or non-trait-based approaches [7,8,15].

Specifically, in our model, response diversity was driving portfolio effects through compensatory effects owing to species’ differential responses to environmental fluctuations (i.e. $CPE_{env}$; see Methods equations (2.9)–(2.11)), thereby modulating the statistical averaging of independent species fluctuations [4]. This mechanism highlights how differences in species’ environmental responses contribute to asynchrony, stabilizing communities by reducing the synchrony of population fluctuations.

A consequence of the large effect of response diversity is that asynchrony in species fluctuations is driven more by the statistical averaging of independent species fluctuations weighted by response diversity (portfolio effects) than by compensatory dynamics emerging from species interactions. This finding is also coherent with previous studies in competitive communities [7,8], but it was less expected to happen in food webs. Trophic interactions are indeed expected to result in more compensatory dynamics resulting from oscillations between predators and prey [53] or prey switching from predators [20,21]. However, pioneering theoretical studies have shown that environmental stochasticity can dampen prey switching [39] and that trophic cascades between trophic levels in species-rich food webs are weak compared to food chains and species-poor food web modules [22]. So, the question of the importance of compensatory dynamics in complex food webs remains an open and interesting avenue for future research.

Population stability (not asynchrony) was found to be the dominant driver of community stability in our food web simulations, through the large effect of environmental stochasticity. These results are in agreement with previous findings in empirical food webs [25] and food web models [26], but contrast with empirical evidence in competitive communities [13]. It suggests fundamental differences between competitive communities and food webs in the relative contributions of population stability and asynchrony to community stability. Our findings suggest that the presence of trophic links contributes to synchronize the species of the community because we found that the food web metrics studied—namely, average trophic level, connectance and average interaction strength—all decrease asynchrony. These results are in line with empirical evidence that predators can synchronize their prey [18,19], and that species pairs involved in trophic interactions are more synchronous than those that are not [25].

A complementary explanation for the relatively lower importance of asynchrony in food webs (compared to single-trophic-level communities) could be related to the biomass structure of food webs. Food webs typically include a larger range of body masses than single-trophic-level assemblages [54]. Such highly uneven biomass distribution can translate in uneven species temporal variability, thereby creating uneven species contributions to asynchrony [12] and limiting portfolio effects and compensatory dynamics in species-rich communities [8].

Investigating in more detail the drivers of community stability, our results suggest that species richness and the network structural properties investigated only have a weak effect on overall community stability. We found that a higher average trophic level leads to higher community stability by increasing the average population stability, confirming previous theoretical [23,24,26] and empirical [25] results. However, this effect disappeared when we simulated community dynamics with equal mortality rates for all species instead of allometric ones (electronic supplementary material, figure S2). This suggests that the higher stability of food webs with higher average trophic levels stems more from their lower mortality rates rather than from their trophic role directly. The overall effect of connectance on overall community stability was found to be quite low, in agreement with previous empirical and theoretical findings [25,26]. Similarly, we found very low effects of the average interaction strength on community stability, which is in apparent disagreement with the predominant role of interaction strength and connectance on stability [2]. We found that average interaction strength had a weak positive effect on population stability, in confirmation with previous theoretical findings aligning with our model assumption that there was no asymmetry of consumer preferences for their prey [55].

### (b) Stability-richness relationships depend on response diversity and food web structure

Our results also allow us to revisit the old question of the relationship between the complexity of ecological communities and their stability [2,56]. We found that response diversity enables the emergence of positive stability-richness relationships, enhanced by average interaction strength and dampened by average trophic level and connectance. In the absence of response diversity, only negative relationships between community stability and species richness are observed. This reinforces the idea that increases in species richness enhance community stability only if the added species have different responses to environmental perturbations, i.e. if there is response diversity [6,29]. Indeed, a community with numerous species which respond differently to environmental change is more likely to buffer a wide array of perturbations [3,28].

Despite its relatively weaker effect on community stability (than response diversity and environmental stochasticity), food web structure interacts strongly with response diversity to determine the sign of stability-richness relationships. Surprisingly, we find that higher average interaction strength enhances positive stability-richness relationships by enhancing asynchrony-richness relationships. Conversely, higher average trophic level and higher connectance both led to negative stability-richness relationships by dampening asynchrony-richness relationships. It would appear that asynchrony becomes too weak to compensate for the simultaneous decrease in population stability induced by increasing species richness.

These results should be understood as the predicted stability-richness relationships if food web structure is kept constant, i.e. if food web structure does not vary with species richness. However, connectance [36] and average trophic level weighted by biomass are documented to decrease with species richness [25], because of energetic and topological constraints [36,57]. Our results add to this body of work by suggesting that communities increasing in species richness without concurrent decreases in connectance and average trophic level should experience reduced stability. Our results resonate with findings that some community structure can lead to negative stability-richness relationships, as previously reported in freshwater food webs [25]. Response diversity might therefore be one of the mechanisms that explains why positive relationships between stability and species richness are so prevalent in empirical settings [12,14], while food web theoretical models that do not often include environmental stochasticity and response diversity typically find negative complexity–stability relationships [40].

### (c) Summary

In conclusion, our study highlights the contrasting effects of response diversity and food web structure on the temporal stability of species-rich communities: response diversity underpins community stability and strongly interacts with food web structure to define stability-richness relationships. In line with the insurance hypothesis [3,48], our results suggest that environmental stochasticity and response diversity are the main mechanisms driving community stability, while food web structural properties are only mediating them. This further aligns with the idea that, in species-rich communities, community structure might be secondary for understanding the generic effects of biodiversity on ecosystem functioning [28,58]. Our results further add to the mounting evidence that population stability is a stronger driver of community stability than asynchrony in food webs, in contrast with what was observed in competitive communities.

The fact that environmental stochasticity acting with response diversity was found to be the main driver of overall community stability raises concerns about the consequences of the current rise in the frequency and severity of environmental extreme events for ecological communities [59,60], which constitutes an increase in environmental stochasticity. Indeed, our results suggest that these changes are unlikely to be compensated by biotic mechanisms alone. Documenting how rapid biodiversity changes [61,62] driven by human pressures [63]—and in particular the reported homogenization [64,65] in community composition—affect response diversity will be key to accurately predict how it will affect, in turn, the stability of communities.

Looking forward, our study brings evidence that asynchrony is predominantly driven by portfolio effects as previously reported in plant communities. However, we expect that some ecological contexts could result in the dominance of compensatory effects owing to species interactions over portfolio effects. An example is the role of the structure of response diversity, since we here considered species responses correlation across whole food web communities. We expect that considering species differential responses to environmental stochasticity within functional groups [14], such as trophic levels, may give more importance to compensatory dynamics because of species interactions relative to the ones related to portfolio effects. The structure of response diversity in food webs has been overlooked so far, but this knowledge is expected to rise with the methodological progress in measuring response diversity [16,17]. Another context to explore is the effect of the distribution of interaction strengths in food webs. Implementing asymmetric distributions of consumer preferences for their prey could also increase the importance of compensatory dynamics owing to species interactions, as strong asymmetry in interaction strength can enhance prey switching behaviour [20,21].

## Data Availability

The code used to run the simulations, analyse them and to write the manuscript is archived on Zenodo: [66]. Supplementary material is available online [67].
